# In silico discovery and evaluation of phytochemicals binding mechanism against human catechol-O-methyltransferase as a putative bioenhancer of L-DOPA therapy in Parkinson disease

**DOI:** 10.5808/gi.20061

**Published:** 2020-12-23

**Authors:** Surya Narayan Rath, Lingaraja Jena, Rajabrata Bhuyan, Nimai Charan Mahanandia, Manorama Patri

**Affiliations:** 1 Department of Bioinformatics, Odisha University of Agriculture and Technology, Bhubaneswar 751003, India; 2 Neurobiology Laboratory, Department of Zoology, School of Life Sciences, Ravenshaw University, Cuttack 753003, India; 3 Centers for Advanced Research & Excellence, Department of Laboratory Oncology, All India Institute of Medical Science, New Delhi 110023, India; 4 Department of Bio-Science and Biotechnology, Banasthali Vidyapeeth (Deemed) University, Banasthali 304022, India; 5 Animal Biotechnology Centre, National Dairy Research Institute, Karnal 132001, India

**Keywords:** inhibitors, L-DOPA, Parkinson disease, phytochemicals, *Withania somnifera*

## Abstract

Levodopa (L-DOPA) therapy is normally practised to treat motor pattern associated with Parkinson disease (PD). Additionally, several inhibitory drugs such as Entacapone and Opicapone are also cosupplemented to protect peripheral inactivation of exogenous L-DOPA (~80%) that occurs due to metabolic activity of the enzyme catechol-O-methyltransferase (COMT). Although, both Entacapone and Opicapone have U.S. Food and Drug Administration approval but regular use of these drugs is associated with high risk of side effects. Thus, authors have focused on *in silico* discovery of phytochemicals and evaluation of their effectiveness against human soluble COMT using virtual screening, molecular docking, drug-like property prediction, generation of pharmacophoric property, and molecular dynamics simulation. Overall, study proposed, nine phytochemicals (withaphysalin D, withaphysalin N, withaferin A, withacnistin, withaphysalin C, withaphysalin O, withanolide B, withasomnine, and withaphysalin F) of plant *Withania somnifera* have strong binding efficiency against human COMT in comparison to both of the drugs i.e., Opicapone and Entacapone, thus may be used as putative bioenhancer in L-DOPA therapy. The present study needs further experimental validation to be used as an adjuvant in PD treatment.

## Introduction

Parkinson disease (PD) pathology is mainly associated with progressive loss or impair function of dopaminergic neurons, occurs as a consequence of chronic inflammation, oxidative stress, deposition of protein aggregates within neurons, depletion of neurotransmitters, abnormal ubiquitination, mitochondrial dysfunction, excitotoxicity of neurons, and disarrangement or damage of the blood-brain barrier (BBB) [1,2]. Although, influences of genetic and environmental factors in PD [3] is well studied but, complete knowledge on disease pathophysiology is still blurred. Motor related symptoms such as tremor, rigidity, and difficulty in coordination of physical movements [4] are common in PD and developed due to depletion of dopamine within an area of midbrain known as substantia nigra pars compacta. Therefore, motor disturbances in PD are treated through administration of exogenous levodopa or L-DOPA (3,4-dihydroxy L-phenylalanine) which provides only symptomatic relief [4]. In addition, co-supplementation of monoamine oxidase B, aldehyde dehydrogenase, and catechol-O-methyltransferase (COMT) inhibitors is also practised to prevent unwanted inactivation of L-DOPA within the brain [5-7].

Human COMT (EC 2.1.1.6, hCOMT) is a magnesium-dependent intracellular enzyme expressed in glial cells and neurons, and associated with diverse spectrum of neurological disorders as well as cancer [8]. hCOMT enzyme metabolizes catecholamines (norepinephrine, epinephrine, and dopamine) by introducing a methyl group from S-adenosyl methionine (SAM) to their catecholamine group [8,9]. COMT exists in two major forms such as membrane-bound (MB) COMT and soluble (S) COMT. The cellular distribution and orientation of MB-COMT on the cellular membrane is controversial [8]. However, S-COMT plays more significant role in peripheral L-DOPA deactivation than central nervous system (CNS) [10]. L-DOPA is not only a precursor of catecholamines but also an important substrate of COMT. Therefore in L-DOPA therapy, COMT inhibitors such as entacapone (Drug Bank ID: DB00494), tolcapone (Drug Bank ID: DB00323), and opicapone (Drug Bank ID: DB11632) have been used as an adjuvant to prolong the availability of L-DOPA [4] within the brain. Entacapone is a U.S. Food and Drug Administration (FDA)‒approved drug that mainly acts peripherally whereas tolcapone acts both peripherally and centrally [11,12].

In PD treatment, lifelong medication is normally recommended by physician to improve the quality of patient’s life [12]. However, continuous uses of synthetic medicines have been reported with adverse effects on hepatic and cardiac health [13,14]. Due to association of serious hepatotoxicity, the drug tolcapone (Drug Bank ID: DB00323) is already withdrawn after investigation. Although, both entacapone and opicapone are FDA-approved drugs, but investigations are still going on to get COMT inhibitors with less side effects. Opicapone is a highly selective, reversible peripheral COMT inhibitor [15,16] but, associated with severe side effects such as dyskinesia, dizziness, dry mouth, and constipation [17]. In this context, several phytochemicals from different neuroprotective plants with antioxidant, anti-inflammatory, antiangiogenic, immune suppressive, anti-apoptosis, protein kinase inhibitor, anticholinesterase, anti‒cyclooxygenase-1 (COX-1) properties have been identified and reported [2,18]. Therefore, it is essential to identify potent drug-like phytochemicals to be used as alternative medicines for the treatment of PD [2,3,18]. The present study has focused on in sillico discovery and assessment of suitable herbal compounds as putative COMT inhibitors which may be experimented for further validation. This study would throw lights on discovery of natural medicine to treat PD patients with no or less risk of side effects.

## Methods

### Extraction and preparation of drug target structure

The X-ray crystallographic structure of hCOMT (PDB ID: 3BWM) attached with its substrate SAM and a substrate analog, 3,5-dinitrocatechol (DNC) was extracted from PDB (Protein Data Bank) (http://www.pdb.org). Initially, all crystallographic water molecules and DNC were removed from the original structure in order to dock herbal compounds into its substrate binding sites. Further, energy minimization of the target structure was performed after adding hydrogen atoms to obtain a properly optimized position of side chain atoms and hydrogen atoms using Discovery Studio 3.5 suite.

### Molecular dynamic simulation of COMT

Molecular dynamic (MD) simulation was performed to study the structural stability of human S-COMT enzyme attached with and without substrates such as SAM and DNC using GROMOS96 54a7 force field of GROMACS 5.0.4 package [19]. The protonation state of the enzyme was achieved at default pH (7.0). Simple point charge water model was embedded in cubic boxes with minimum edge distance of 10 Å from the protein surface to solvate the systems. Further, electrical neutral state was attained by adding chlorine ions and replacement of water [20]. Subsequently, steepest descent energy minimization was carried out until reaching to a force tolerance of 1,000 kJ/mol. Afterwards, systems were equilibrated at 300 K for 100 ps (NVT) by restraining all heavy atoms of protein backbone chain, followed by 100 ps of pressure equilibrium (NPT). During NPT equilibration, all of the restraints were withdrawn. Velocity rescale thermostat [21] was used with a time constant (τT) of 0.1 ps for temperature coupling. At the same time, isotropic Parrinello-Rahman barostat (1981) was set to 1.0 bar in all directions with a time constant (τP) of 2.0 ps at the time of pressure coupling. Particle mesh Ewald method [22] was employed to take care of long-range Coulomb interactions. Similarly, the linear constraint solver (LINCS) algorithm [23] was used to restrict all bond lengths for a time step of 2 fs. During MD simulation, Van der Waals forces and Coulomb interactions cut-off distances were maintained at 1.0 nm of each. Each MD simulations were performed independently for a time period of 50 ns for all of the systems (COMT with and without SAM and DNC) [24].

### Inspection of ligand binding site

The optimized structure of hCOMT was subsequently inspected to identify apposite active and functional site, where substrate normally binds to initiate its proper biochemical function. The amino acids strongly interacting with the substrate analog DNC and the ion Mg^2+^ were considered as active site for ligand interaction.

### Retrieval and preparation of ligand structures

Based on literature evidence, we found total 80 numbers of phytochemicals with anti-PD properties from different medicinal plants [2,18,24]. Three-dimensional structures of these compounds were extracted from PubChem (https://pubchem.ncbi.nlm.nih.gov/) database in SDF format and were converted to PDB format using Open Babel [25] to carry out further *in silico* studies. Structural geometry optimization and protonation state of these ligands were achieved using Discovery Studio 3.5 suite.

### Drug-like property prediction

Molinspiration (http://www.molinspiration.com/) web server was used to predict the drug-like property of selected phytochemicals. It accepts ligand structure in SMILES (Simplified molecular-input line-entry system) format and predicts its bioactivity and pharmacokinetics properties following Lipinski’s rule of five [26].

### Screening of ligands

Selected natural compounds were screened computationally against complex structure of hCOMT and SAM in order to identify efficient ligand using PyRx0.8 tool (https://pyrx.sourceforge.io/). PyRx 0.8 is an open source tool [27], used to screen libraries of compounds against potential drug target [24,28]. During virtual screening (VS) a grid of 30, 30, 30 Å in x, y, z direction was centred on drug-binding pocket of hCOMT crystal structure using AutoDock Vina [29] and PyRx 0.8 [27].

### Molecular docking

Molecular docking was performed to validate the efficiency of selected natural compounds obtained from VS and drug-like property prediction. During docking, two FDA-approved anti-Parkinson COMT inhibitor drugs such as opicapone (DB11632), and entacapone (DB00494) were also included to compare their binding affinity with selected natural ligands. Molecular docking was performed using AutoDock 4.2 (http://autodock.scripps.edu/) and Auto-Dock Tools 4 tool [30]. Each ligand was docked independently with the enzyme COMT. During docking and further studies, Mg^2+^ ion was kept intact in its position. The receptor and ligands were prepared using ADT tool [30]. Kollman charges and polar hydrogen atoms were added to the enzyme structure. Gasteiger partial charge was applied and nonpolar hydrogen atoms were merged within ligand structures. Both receptor and ligands were converted to pdbqt format before docking. A virtual grid box was set around the drug-binding cavity of the target structure with size of 30, 30, 30 Å in x, y, z direction along with spacing of 0.375 Å. Semi-flexible docking was performed by keeping the protein as rigid and allowing ligands to move within the binding cavity. Lamarckian genetic algorithm was employed to perform molecular docking. During the docking process, a maximum of 20 conformers was considered for each docking with 25,000,000 energy evaluation steps. Subsequently, all binding poses of each docking were studied and most energetically as well as geometrically favorable conformation for each independent run was selected for further study. Finally, 2D and 3D view of atomic interaction between best-docked complexes were achieved using Discovery Studio 3.5 and PyMol molecular graphics (http://www.pymol.org) tool, respectively.

### MD simulation of COMT in the presence of SAM and natural ligands

To confirm the stability and efficacy of natural ligands fitted into the active pocket of COMT and in the presence of SAM, MD simulation of protein-ligand complex [24] was performed for 10 suitable phytochemicals. PRODRG [31] web server was used to prepare each ligand topology. Rest of the protocol was same as described above. Ten independent MD run were performed for 50 ns time period. Trajectories of all 10 simulations were saved in 10 fs interval. Microsoft Excel was used to plot graphs from the produced results.

### Prediction of pharmacophoric features

Knowledge on different pharmacophoric properties of a lead molecule has a vital role in computer aided drug design (CADD). Presence of few chemical features such as aromatic ring (AR), hydrogen bond donor (HBD), hydrogen bond acceptor (HBA), hydrophobic property (HY) were predicted for all 10 suitable phytochemicals using ZINC Pharmer (http://zincpharmer.csb.pitt.edu/) web server.

## Results

### COMT identified as a significant drug target in PD

**Fig. 1.**
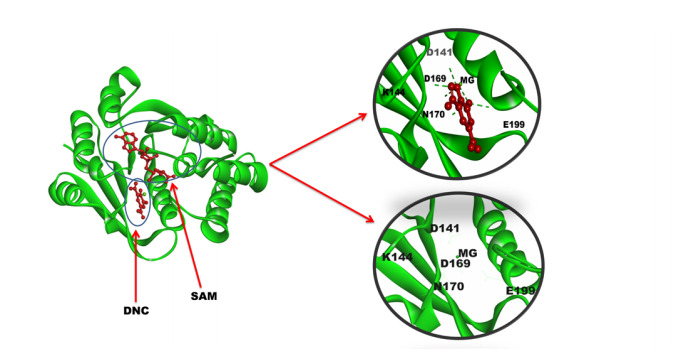
Crystal structure of human catechol-O-methyltransferase (COMT) (PDB ID: 3BWM, chain A) enzyme with substrate S-adenosyl methionine and 3,5-dinitrocatechol (DNC) (left). Amino acids found interacting with DNC and Mg^2+^ ion within the active pocket of COMT are deciphered in the right side.

Possible conversion of exogenous L-DOPA to 3-O-methyldopa in both peripheral and cerebral system occurs due to the metabolic activity of COMT enzyme, is a major concern in PD treatment. In this connection, discovery and synthesis of chemical inhibitors has been aided by several solved structures of both rat and hCOMT enzyme available at PDB. Different crystal structures of human and rat COMT enzyme were observed with diverse substrate specificity and conformation. In addition, significant decrease in protein level as well enzymatic activity of COMT has been reported in association of a valine-methionine polymorphism at position 108 of hCOMT [32]. Here, we have retrieved the soluble form of hCOMT structure (PDB ID: 3BWM, chain A, length: 214, 1.98Å resolution) connected with substrates SAM and DNC. After inspection, it was identified, amino acid residues such as ASP141, LYS144, ASP169, ASN170, GLU199, and Mg^2+^ were strongly interacting with the substrate analog DNC, thus considered as active drug-binding site for further study (Fig. 1).

### Validation of COMT stability by MD simulation in the presence and absence of substrates

**Fig. 2.**
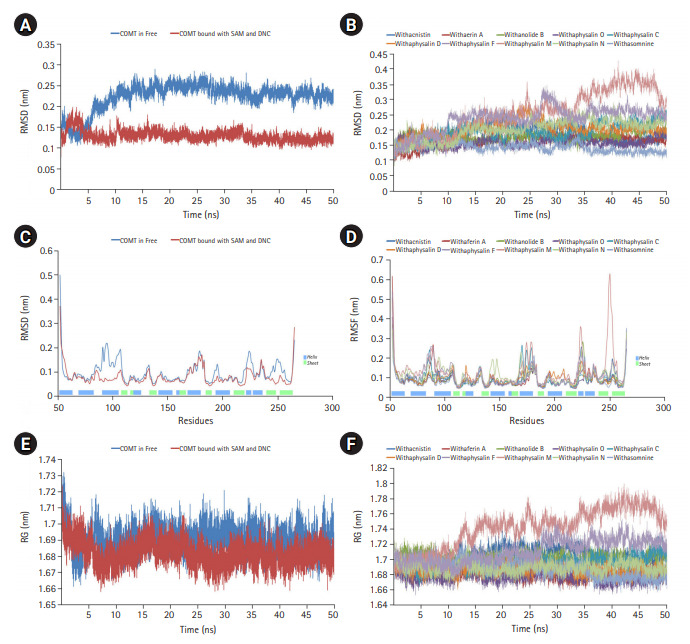
Molecular dynamics simulation (MD) plots of catechol-O-methyltransferase (COMT) enzyme attached with and without S-adenosylmethionine (SAM) and a substrate analog, 3,5-dinitrocatechol (DNC): root mean square deviation (RMSD) plot of backbone (A), root mean square fluctuation (RMSF) plot for residue wise fluctuation (B), and radius of gyration (RG) plot for overall compactness of the system (C). MD simulation plots of COMT enzyme in complex with 10 different phytochemicals of plant *Withania somnifera*: RMSD plot of backbone (D), RMSF plot for residue wise fluctuation (E), and RG plot for overall compactness of the system in presence of phytochemicals (F).

Macromolecules are not static in nature, so their movement causes structural fluctuation with varying energies which may affect their relevant functional phenomena. MD simulation is the one and only computational method to study the functional behavior of biological molecules such as protein or enzyme in different thermodynamical condition with respect to time scales [24,33]. Here, we performed MD simulation to discover the time dependant structural fluctuation and functional stability of the enzyme human S-COMT (PDB ID: 3BWM) in the presence and absence of substrate SAM and the substrate analog DNC. Both of the MD run was performed independently for 50 ns using GROMOS96 54a7 force field of GROMACS 5.0.4 package [18,24,33]. Overall structural consistency and stability of the backbone folding pattern was observed from root mean square deviation (RMSD) plot (Fig. 2A) in both of the systems after around ~10 ns. However, the system with substrates (SAM and DNC) was achieved the stability more quickly with an average deviation of 0.13 nm from the starting structure. Similarly, the overall root mean square fluctuation (RMSF) of COMT with SAM and DNC showed less flexibility as compared to the system in the absence of substrates (Fig. 2B). The overall packing of the systems was justified from the radius of gyration (RG) plot (Fig. 2C). As per RG plot, the packing of atoms in proteins in both of the systems (with and without substrates) were almost same (difference with only ~0.03 nm) throughout the simulation period of 50 ns (Fig. 2C). The overall MD simulation of COMT in the presence of SAM and DNC proved to be more stable than the enzyme COMT alone (Fig. 2A‒2C).

### Selection of suitable phytochemicals

Plant’s crude extracts and plant-oriented natural compounds from several medicinal plants have been studied to explore their neuroprotective effect using different *in silico* and in vivo models [2,3,18,24,34-36]. Therefore, *in silico* identification of phytochemicals may be useful to discover suitable natural inhibitors against the PD drug target COMT. In the present study, total 80 numbers of phytochemicals (Supplemental Table 1) with medicinal properties were selected from the literature [2,18].

### Pharmacokinetic properties of proposed drug-like phytochemicals

Determination of pharmacokinetic profile such as absorption, distribution, metabolism, excretion, and toxicology is crucial to verify the suitability of any small compound to be used as a lead molecule [37]. According to Lipinski’s rule of five a lead molecule should have ≤10 HBA, ≤5 HBD, ≤500 molecular weight, ≤5 octanol/water partition coefficient (miLogP), ≤90 Å square topological polar surface area (TPSA). It is considered, reduction in bioactivity of a lead molecule may occur due to violation of any of these two properties [26]. Here, the prediction proposed only 63 phytochemicals with good pharmacokinetic profile (Supplemental Table 2). Again, as of Lipinski’s rule of five [26], the permeability through cell membrane for a lead compound is evaluated through its computed TPSA value. So, compound with TPSA value greater than 140 angstroms squared tend to be poor at permeating cell membranes [38]. But, in case of CNS-related drugs, the TPSA value less than 90 Å squared is mostly acceptable which indicates their ability to penetrate through the cell membrane as well as BBB [39]. Therefore, after ADMET analysis, it is recommended, out of 63 compounds (Supplemental Table 2), only 39 (Supplemental Table 3) bioactive compounds have the ability to cross the BBB, thus may be useful to be used as CNS drugs. However, total 17 compounds were strongly violated one or two Lipinski’s rule (Supplemental Table 2), therefore discarded from further study.

### VS recommended efficient natural ligands

VS of ligands have been utilized successfully as an effective *in silico* technique for filtering out potential ligands against appropriate drug target [24,27,28,37]. It is an economical and time-saving approach and possibly helps experimental procedure to increase the success rate in drug discovery. Here, site-directed VS was performed for 63 previously studied phytochemicals with good pharmacokinetic properties (Supplemental Table 2). As, COMT enzyme has the ability to degrade L-DOPA both peripherally and centrally [8-10,32] therefore, all of these 63 natural ligands (Supplemental Table 2) were screened to discover compounds with potential binding affinity against the drug target. According to VS result, four phytochemicals such as withaphysalin M, withaphysalin N, withaphysalin F, and withaphysalin O of plant *Withania somnifera* were showed better binding energy than rest others (Supplemental Table 4). However, on the basis of suitable binding affinity and pharmacokinetic profile 15 natural compounds (Table 1) of *W. somnifera* plant were subjected for further validation using molecular docking study.

### Molecular docking confirmed the binding efficiency of *W. somnifera* phytochemicals against COMT

**Fig. 3.**
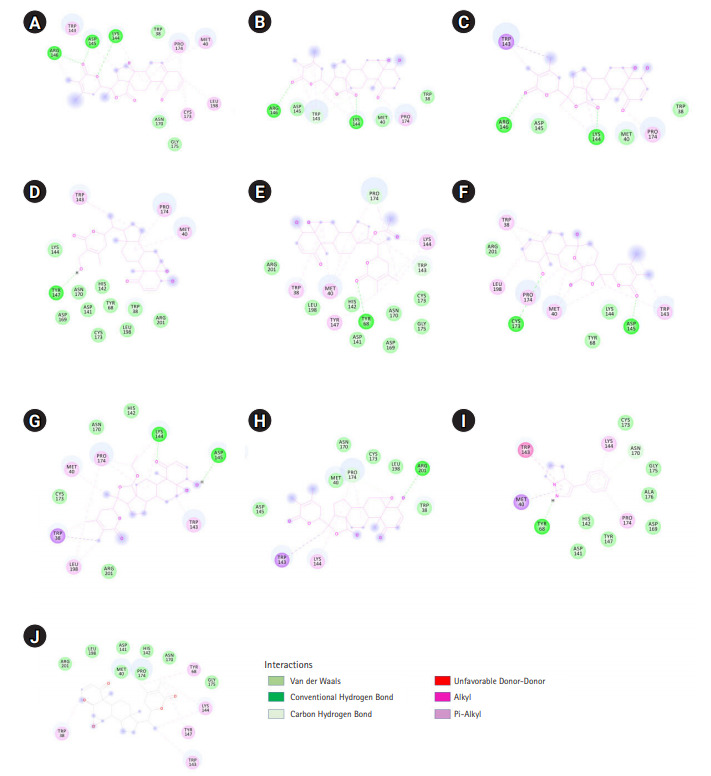
The 2D view of close amino acid residues participated in h-bond, Van der Waals interaction, and Pi-Alkyl interaction with phytochemicals within the active pocket of human catechol-O-methyltransferase (COMT) are represented: withaphysalin D (A), withaphysalin N (B), withaphysalin M (C), withaferinA (D), withacnistin (E), withaphysalin C (F), withaphysalin O (G), withaphysalin F (H), withasomnine (I), and withanolide B (J).

**Fig. 4.**
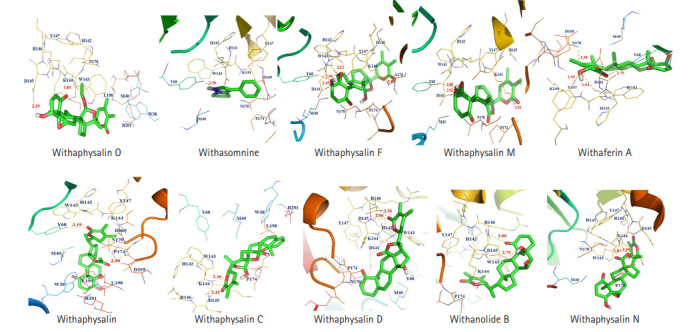
Atomic interaction including h-bond between 10 phytochemicals (withaphysalin O, withasomnine, withaphysalin F, withaphysalin M, withaferinA, withacnistin, withaphysalin C, withaphysalin D, withanolideB, and withaphysalin N) and human catechol-O-methyltransferase enzyme are depicted.

Total 15 suitable phytochemicals (Table 1) of plant *W. somnifera* were docked into the drug-binding pocket of human S-COMT. The binding energy of protein-ligand complex resulted from molecular docking was compared with VS score of each compound and reported (Table 2). It was confirmed all of these 15 phytochemicals have potential binding efficiency against hCOMT (Table 2). In addition, 10 natural compounds such as withaphysalin M (‒7.42 kcal/mol), withaphysalin N (‒7.24 kcal/mol), withaphysalin F (‒6.48 kcal/mol), withaphysalin O (‒6.78 kcal/mol), withaphysalin C (‒6.85 kcal/mol), withaphysalin D (‒7.84 kcal/mol), withanolide B (‒7.63 kcal/mol), withaferin A (‒7.53 kcal/mol), withacnistin (‒7.13 kcal/mol), and withasomnine (‒6.09 kcal/mol) were showed consistency in binding energy scores (Table 2) with VS scores which advocated for their reliability in binding against COMT. Further, two FDA-approved drugs such as opicapone (DB11632), and entacapone (DB00494) were docked within the drug-binding site of COMT. Interestingly, eight phytochemicals such as withaphysalin D (‒7.84 kcal/mol; KI: 1.8 μM), withanolide B (‒7.63 kcal/mol; KI: 2.54 μM), withaferin A (‒7.53 kcal/mol; KI: 3.03 μM), withaphysalin M (‒7.42 kcal/mol; KI: 3.67 μM), withaphysalin N (‒7.24 kcal/mol; KI: 4.93 μM), withacnistin (‒7.13 kcal/mol, KI: 5.92 μM), withaphysalin C (‒6.85 kcal/mol, KI: 9.56 μM), withaphysalin O (6.78 kcal/mol; KI: 10.76 μM) were docked with better binding affinity and inhibition constant (Table 3) than both of the drugs i.e., opicapone (‒6.74 kcal/mol; KI: 11.41 μM), entacapone (‒6.34 kcal/mol; KI: 22.55 μM). However, rest five phytochemicals namely cuscohygrine (‒6.51 kcal/mol; KI: 16.96 μM), withaphysalin F (‒6.48 kcal/mol; KI: 17.83 μM), anaferine (‒6.33 kcal/mol; KI: 23.06 μM), pelletierine (‒6.31 kcal/mol; KI: 23.5 μM), and withasomnine (‒6.09 kcal/mol; KI: 34.35 μM) were appeared as close binding competitors of both of the drugs (Table 3). Again, the presence of ample numbers of amino acid residues in hydrogen bond formation, Van der Waals interaction, and Pi-Alkyl interaction within active site of hCOMT enzyme also established significant interaction of the phytochemicals with COMT (Fig. 3). Again, participation of strong polar interactions (distance ≤3Å) between hCOMT and phytochemicals (Table 4, Fig. 4) of plant *W. somnifera* were perceived in favor of the above observation. To its support, few amino acids such as Tyr 68 (withaphysalin M, withaferinA, withacnistin, withasomnine, withaphysalin F), Lys144 (withaphysalin N, withaphysalin M, withaphysalin C, withaphysalin O, withaphysalin F), Asp145 (withaphysalin D, withanolide B, withaphysalin C, withaphysalin O), and Arg146 (withaphysalin D, withanolide B) were identified as commonly participated in polar interaction within the binding cavity (distance ≤3.5Å) of hCOMT (Table 4, Fig. 4) enzyme. However, the overall study confirmed about their strong atomic interaction with hCOMT consequently, subjected for MD simulation.

### MD simulation established structural stability of COMT-phytochemical complex in presence of SAM

**Fig. 5.**
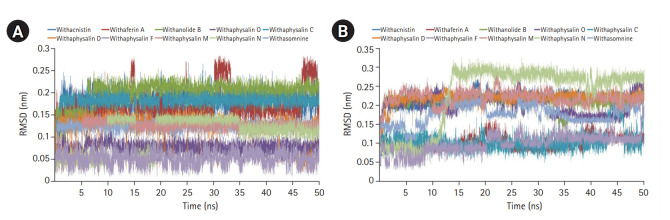
Root mean square deviation plot of all 10 individual phytochemicals (A) and S-adenosylmethionine (SAM) in their respective enzyme-ligand-SAM complex (B) are depicted.

On the basis of recommendation of all previous observations, 10 phytochemicals (withaphysalin M, withaphysalin N, withaphysalin F, withaphysalin O, withaphysalin C, withaphysalin D, withanolide B, withaferin A, withacnistin, and withasomnine) of plant *W. somnifera* were appeared to have possible impact to block the active site of human S-COMT, insisted authors to perform MD simulation of the enzyme (PDB ID: 3BWM) in the presence of these phytochemicals along with its natural substrate SAM to observe its structural and functional behavior in complex form. Ten independent MD run were performed for protein-ligand complex up to 50 ns time scale. From the RMSD plot of backbone atomic structure, it was identified, hCOMT attached with different phytochemicals were quite consistent after around ~30 ns, suggesting the better stability of the enzyme except in one case, i.e., the COMT and withaphysalin M complex (Fig. 2D). In this case, significant fluctuation in RMSD plot was observed after around ~34 ns which continued till the end of 50 ns MD simulation, indicated about the instability of COMT and withaphysalin M complex (Fig. 2D). Similar type of observation was perceived from the RMSF plot (Fig. 2E). Minor fluctuation in amino acid residual positions was noticed for all COMT-phytochemical complexes except withaphysalin M which pointed out the binding stability of all nine natural compounds (Fig. 2E) of *W. somnifera* plant. Higher fluctuations in amino acids of COMT were observed near the regions having no specific secondary structure. Similarly, the overall packing of COMT enzyme was found quite stable and compact throughout the simulation period of 50 ns in all nine protein-ligand complexes except withaphysalin M as plotted in RG plot (Fig. 2F). Furthermore, to strengthen this hypothesis, RMSD plot of different phytochemicals and SAM from all of the MD systems were plotted (Fig. 5A and 5B). Quite satisfactory observation was noticed in case of all natural compounds (Fig. 5A) and SAM (Fig. 5B) in their respective enzyme-ligand-SAM complex within time scale of 50 ns MD simulation. Overall, MD simulation results discovered, out of 10 only nine phytochemicals of plant *W. somnifera* have potential binding stability against the soluble hCOMT enzyme.

### Presence of pharamacophoric features advocated for phytochemicals efficacy

**Fig. 6.**
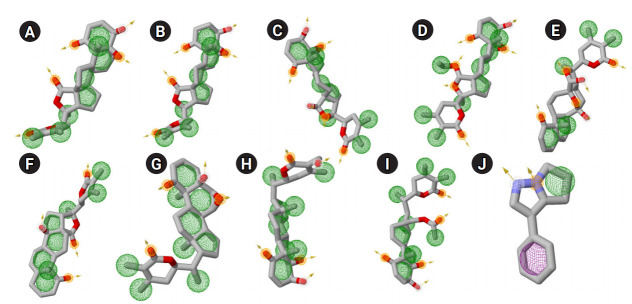
Predicted 3D pharmacophoric features of 10 different phytochemicals are represented: withaphysalin M (A), withaphysalin N (B), withaphysalin F (C), withaphysalin O (D), withaphysalin C (E), withaphysalin D (F), withanolide B (G), withaferin A (H), withacnistin (I), and withasomnine (J). The aromatic ring, hydrophobic feature, hydrogen bond donor and hydrogen bond acceptors are shown in purple, green, white and gold spheres, respectively. The arrows presented here for the constraint direction.

Pharmacophore based ligand discovery has a critical role in CADD [37,40]. Therefore, identification of important chemical features such as the presence of AR, hydrophobic feature (HY), HBD, and HBA are necessary to confirm the effect of interaction between a lead molecule and the drug target. Presence of above pharmacophoric properties was discovered in functional groups of all 10 suitable phytochemicals (Table 5, Fig. 6) and thus, strongly recommended for their effectiveness and sensible binding interaction against the PD drug target COMT.

## Discussion

Disturbance of motor activity has been perceived as a preliminary symptom [4] in PD and is usually treated through administration of L-DOPA [12]. COMT plays a significant role in the metabolism of L-DOPA, thus inactivates exogenous L-DOPA both in peripheral and CNS. Therefore, few COMT inhibitor drugs such as Opicapone and Entacapone are cosupplemented with L-DOPA to maintain the dopamine level within CNS [4,10,12]. On the contrary, the long-term uses of these medicines are associated with the risk of patient’s cardiac and hepatic health [17]. In this context, possible use of plant-oriented natural inhibitors has received growing interest of scientist globally. Since ages, many pant derived natural compounds or phytochemicals have been known to be effective against neurological disorders due to the presence of their antioxidant, anti-inflammatory, antiangiogenic, immune suppressive, anti-apoptosis, protein kinase inhibitor, anticholinesterase, anti‒COX-1 properties [2,18,24]. The rationale of this approach is established through several in silico, in vitro, in vivo, and preclinical studies [2,3,18,24,34-36,41]. Additionally, the use of phytochemicals offers advantages over synthetic drugs such as no or minimal side effects.

The immense importance of different neuroprotective natural compounds encouraged authors to investigate binding stability and suitability as putative inhibitors against PD drug target COMT. Based on literature evidence, structures of 80 natural compounds [2,18,24] were retrieved from the public repository and subjected to verify their drug-like property. Pharmacokinetics of a potential inhibitor depends on its good drug-like properties [37] which were verified by following the Lipinski’s rule of five [26]. Upon verification, suitable drug-like property was confirmed in case of 63 phytochemicals (Supplemental Table 2) therefore structures of those phytochemicals were virtually screened against crystal structure of hCOMT within its known drug-binding site. VS technique has been revealed as a promising in silico procedure to identify potential lead compounds against any drug target [24,27,28,33,37]. VS result was proposed four phytochemicals such as withaphysalin M, withaphysalin N, withaphysalin F, and withaphysalin O (Supplemental Table 4) of plant *W. somnifera* with strong binding affinity against hCOMT. However, on the basis of suitable binding affinity and drug-like properties total 15 phytochemicals (Table 1) were selected for further study. Further, molecular docking was performed to confirm binding affinity and binding pattern of these 15 natural compounds. Comparative analysis of VS and docking results was revealed 10 natural compounds (withaphysalin M, withaphysalin N, withaphysalin F, withaphysalin O, withaphysalin C, withaphysalin D, withanolide B, withaferin A, withacnistin, and withasomnine) as suitable due to their consistency in binding scores (Table 2). Further to compare the binding affinity of natural compounds with synthetic COMT inhibitors two FDA-approved drugs namely opicapone (DB11632), and entacapone (DB00494) were also docked within the drug-binding site of COMT. Interestingly, better binding affinity and inhibition constant was found in case of eight phytochemicals (withaphysalin D, withanolideB, withaferinA, withaphysalin M, withaphysalin N, withacnistin, withaphysalin C, and withaphysalin O) than both of the drugs (Table 3) which confirmed their efficacy. To its support, interaction analysis was suggested for significant binding pattern between selected 10 natural compounds (Table 2) of plant *W. somnifera* due to the presence of good numbers of strong hydrogen bond (distance ≤3Å), Van der Waals interaction, and Pi-Alkyl interaction within active site of hCOMT enzyme (Table 4, Figs. 3 and 4).

In order to assess the stability and conformational changes in COMT upon binding of these 10 suitable phytochemicals of plant *W. somnifera* MD simulation was performed for 50ns. The values of RMSD, RMSF, and RG plot suggested the binding of all of these nine phytochemicals of plant *W. somnifera* except withaphysalin M stabilized the COMT structure in presence of SAM (Fig. 2D‒2F) without any conformational shift. However, several random fluctuations were seen initially, but no conformational switching was observed during entire simulation period (Fig. 2D‒2F). Notably, RMSD and RMSF values of SAM and all of these 10 phytochemicals were found quite satisfactory in their respective enzyme-ligand-SAM complex (Fig. 5) within 50 ns MD simulation. In addition, the pharmacophoric features of all of these phytochemicals found suitable to be used as lead compounds against PD drug target COMT (Table 5, Fig. 6). The overall analysis hypothesized, all of these nine phyochemicals (withaphysalin N, withaphysalin F, withaphysalin O, withaphysalin C, withaphysalin D, withanolideB, withaferinA, withacnistin, and withasomnine) of plant *W. somnifera* have potential binding efficiency and may be used as putative inhibitors against PD drug target COMT.

In conclusion, the present *in silico* study discovered, total of nine phytochemicals (withaphysalin D, withaphysalin N, withaferinA, withacnistin, withaphysalin C, withaphysalin O, withanolide B, withasomnine, withaphysalin F) of plant *W. somnifera* (ashwagandha) with good pharmacokinetic profile, pharmacophoric features and stable binding potentiality against hCOMT enzyme. Thus, it is hypothesized that these phytochemicals may be used as putative bioenhancer in L-DOPA treatment. The present study would throw lights on discovery of natural inhibitors against COMT as an alternative treatment of PD and may be further extended for experimental validation in the future.

## Figures and Tables

**Table 1.** Binding energy scores of 15 drug-like phytochemicals of plant *Withania somnifera* resulted from virtual screening against human COMT enzyme

No.	Ligand	Binding energy score	Drug likeness (Lipinski’s rule of five)	BBB permeant
1	Withaphysalin M	‒10.3	Suitable	No
2	Withaphysalin N	‒10.3	Suitable	No
3	Withaphysalin F	‒9.9	Suitable	No
4	Withaphysalin O	‒9.5	Suitable	No
5	Withaphysalin C	‒5.8	Suitable	No
6	Withaphysalin D	‒5.3	Suitable	Yes
7	Withanolide B	‒6.0	Suitable	Yes
8	Withaferin A	‒6.1	Suitable	No
9	Withacnistin	‒5.9	Suitable	No
10	Withasomnine	‒5.3	Suitable	Yes
11	Anaferine	‒4.6	Suitable	Yes
12	Calystegine B2	‒5.2	Suitable	No
13	Cuscohygrine	‒4.2	Suitable	Yes
14	Pelletierine	‒3.9	Suitable	Yes
15	Tropine	‒4.0	Suitable	Yes

COMT, catechol-O-methyltransferase; BBB, blood-brain barrier.

**Table 2.** Comparative account of binding energy scores resulted from virtual screening and molecular docking between 15 phytochemicals of *Withania somnifera* plant and human COMT enzyme

No.	Ligand	Binding energy score	
		Active site based virtual screening (kcal/mol)	Molecular docking (kcal/mol)
1	Withaphysalin M	‒10.3	‒7.42
2	Withaphysalin N	‒10.3	‒7.24
3	Withaphysalin F	‒9.9	‒6.48
4	Withaphysalin O	‒9.5	‒6.78
5	Withaphysalin C	‒5.8	‒6.85
6	Withaphysalin D	‒5.3	‒7.84
7	Withanolide B	‒6.0	‒7.63
8	Withaferin A	‒6.1	‒7.53
9	Withacnistin	‒5.9	‒7.13
10	Withasomnine	‒5.3	‒6.09
11	Anaferine	‒4.6	‒6.33
12	Calystegine B2	‒5.2	‒4.98
13	Cuscohygrine	‒4.2	‒6.51
14	Pelletierine	‒3.9	‒6.31
15	Tropine	‒4.0	‒4.94

COMT, catechol-O-methyltransferase.

**Table 3.** Docking scores of 15 phytochemicals and two synthetic inhibitors (opicapone and entacapone) against human COMT resulted from molecular docking

No.	Ligand (phytochemical/drug)	Binding energy score (kcal/mol)	Inhibition constant (μM)
1	Withaphysalin D	-7.84	1.8
2	Withanolide B	-7.63	2.54
3	Withaferin A	-7.53	3.03
4	Withaphysalin M	-7.42	3.67
5	Withaphysalin N	-7.24	4.93
6	Withacnistin	-7.13	5.92
7	Withaphysalin C	-6.85	9.56
8	Withaphysalin O	-6.78	10.76
9	Opicapone^a^	-6.74	11.41
10	Cuscohygrine	-6.51	16.96
11	Withaphysalin F	-6.48	17.83
12	Entacapone^a^	-6.34	22.55
13	Anaferine	-6.33	23.06
14	Pelletierine	-6.31	23.5
15	Withasomnine	-6.09	34.35
16	Calystegine B2	-4.98	224.43
17	Tropine	-4.94	240.97

COMT, catechol-O-methyltransferase.

a Drug compounds.

**Table 4.** Strong atomic interaction predicted between 10 phytochemicals of *Withania somnifera* plant and human COMT enzyme (distance ≤3.5 Å)

No.	Phytochemical	Predicted amino acid residues within active site of COMT (distance ≤3 Å)	Predicted h-bond residues	Bond	Distance between atoms (Å)
1	Withaphysalin D	Met 40, Asp 141,His 142, Trp 143, Lys144, Asp145, Arg 146, Tyr 147, Asn 170, Pro 174	Asp145	HN---O	2.66
			Arg146	HN---O	3.33
					
2	Withanolide B	His 142, Trp 143, Lys 144, Asp145, Arg 146, Tyr 147, Pro 174	Asp145	O---HO	2.75
			Arg146	HN---O	3.00
					
3	Withaphysalin N	Met 40, His 142, Trp 143, Lys 144, Asp145, Arg 146, Tyr 147, Asn170, Pro 174	Lys144	HN---O	2.95
				HN---O	2.87
4	Withaphysalin M	Met 40, Tyr 68, Asp 141, His 142, Trp 143, Lys144, Asp145, Tyr 147, Asn 170, Pro 174, Ala 176	Tyr 68	O---HO	2.80
			Asp 141	O---HO	2.53
			Lys144	N---O	2.93
					
5	Withaferin A	Met 40, Tyr 68, Asp 141, His 142, Trp 143, Tyr 147, Asp 169, Asn 170	Tyr 68	O---O	2.75
			His 142	O---HO	2.94
			Tyr147	O---HO	2.95
			Asn 170	N---OH	3.38
					
6	Withacnistin	Trp 38, Met 40, Tyr 68, His 142, Trp 143, Lys 144, Tyr 147, Asp 169, Asn 170, Pro 174, Leu 198, Glu 199, Arg 201, Asp 205	Tyr68	OH---O	3.19
			Pro 174	N---O	3.30
7	Withaphysalin C	Trp 38, Met 40, Tyr 68, His 142, Trp 143, Lys 144, Asp 145, Arg 146, Pro 174, Leu 198, Arg 201	Lys 144	HN---O	2.16
			Asp145	HN---O	2.11
					
8	Withaphysalin O	Trp 38, Met 40, His 142, Trp 143, Lys 144, Asp 145, Arg 146, Tyr 147, Asn 170, Leu 198, Arg 201	Lys144	NH---O	1.81
			Asp145	O---HO	2.19
					
9	Withasomnine	Met 40, Tyr 68, Asp 141, His 142, Trp 143, Lys 144, Tyr 147, Asp 169, Asn 170, Cys 173, Pro 174, Gly 175, Ala 176	Tyr68	O---NH	2.58
10	Withaphysalin F	Met 40, Tyr 68, Asp 141, His 142, Trp 143, Lys 144, Asp 145, Tyr 147, Asn 170, Pro 174, Ala 176	Tyr 68	O---OH	2.66
			Asp141	O---OH	2.45
			His142	O---HO	3.13
			Lys144	O---N	3.00

COMT, catechol-O-methyltransferase.

**Table 5.** Pharmacophoric aspects of 10 *Withania somnifera* plant phyochemicals were summarized

No.	Phytochemical	AR	HBD	HBA	HY
1	Withaphysalin M	0	1	4	8
2	Withaphysalin N	0	1	4	8
3	Withaphysalin F	0	2	4	8
4	Withaphysalin O	0	1	5	9
5	Withaphysalin C	0	2	4	6
6	Withaphysalin D	0	1	3	8
7	Withanolide B	0	1	3	9
8	Withaferin A	0	2	3	8
9	Withacnistin	0	1	4	9
10	Withasomnine	1	1	1	1

AR, aromatic ring; HBD, hydrogen bond donner; HBA, hydrogen bond acceptor; HY, hydrophobic feature.

## Supplementary Materials

Supplementary Table 1. 3D structure of total eighty phytochemicals extracted from PubChem database and reported along with their medicinal values

Supplementary Table 2. Predicted drug likeness properties of total eighty phytochemicals

Supplementary Table 3. Thirty-nine phytochemicals permeable through blood-brain barrier and their drug likeness properties

Supplementary Table 4. Binding energy scores of 63 drug-like phytochemicals resulted from virtual screening against human COMT enzyme
