# Supplementary material for: In silico discovery and evaluation of phytochemicals binding mechanism against human catechol-O-methyltransferase as a putative bioenhancer of L-DOPA therapy in Parkinson disease
Source: Genomics Inform. 2020 Dec 23;19(1):e7. doi: 10.5808/gi.20061 (PMC8042297; doi:10.5808/gi.20061)
Supplement: Supplementary Table 1. — 3D structure of total eighty phytochemicals extracted from PubChem database and reported along with their medicinal values [file gi-20061suppl1.docx]

**Supplementary Table 1.** 3D structure of total eighty phytochemicals extracted from PubChem database and reported along with their medicinal values

| **No.** | **Natural compounds** | **Pub Chem**  **CID** | **MW (g/mol)** | **Plant name** | **Property** |
| --- | --- | --- | --- | --- | --- |
| 1 | Withaferin A | 265237 | 470.60 | *Withania somnifera*  (ashwagandha) | Antioxidant,  anti-inflammatory |
| 2 | Withanolide A | 11294368 | 470.60 |  |  |
| 3 | Withanolide B | 14236711 | 454.60 |  |  |
| 4 | Withanolide D | 161671 | 470.60 |  |  |
| 5 | Withaphysalin C | 191450 | 484.58 |  |  |
| 6 | Withaphysalin D | 180584 | 466.57 |  |  |
| 7 | Withaphysalin F | 44566968 | 484.58 |  |  |
| 8 | Withaphysalin M | 10096775 | 482.57 |  |  |
| 9 | Withaphysalin N | 11752064 | 484.58 |  |  |
| 10 | Withaphysalin O | 10436447 | 512.64 |  |  |
| 11 | Withacnistin | 54606507 | 512.64 |  |  |
| 12 | Stigmasterol | 5280794 | 412.70 |  |  |
| 13 | Tropine | 449293 | 141.21 |  |  |
| 14 | Anaferine | 443143 | 224.34 |  |  |
| 15 | Withasomnine | 442877 | 184.24 |  |  |
| 16 | Chlorogenic Acid | 1794427 | 354.31 |  |  |
| 17 | Cuscohygrine | 441070 | 224.34 |  |  |
| 18 | Pelletierine | 92987 | 141.21 |  |  |
| 19 | Calystegine B2 | 124434 | 175.18 |  |  |
| 20 | Dulcitol | 11850 | 182.17 |  |  |
| 21 | Withanolide E | 301751 | 486.605 |  |  |
| 22 | Withafastuosin E | 387980 | 488.621 |  |  |
| 23 | Scopoletin | 5280460 | 192.17 |  |  |
| 24 | Withanone | 21679027 | 470.60 |  |  |
| 25 | Genistein | 5280961 | 270.24 | *Glycine max*  (soybean) | Antioxidant, anti-inflammatory, antiangiogenic, immune suppressive, protein kinase inhibitor |
| 26 | Daidzin | 107971 | 416.38 |  |  |
| 27 | 24-Epibrassinolide | 102515299 | 480.68 | *Vicia faba*  (broad bean) | Antioxidant, anti-inflammatory, anti- apoptosis |
| 28 | 28-Homocastasterone | 5487654 | 478.71 |  |  |
| 29 | Quercetin | 5280343 | 302.23 | *Marsilea quadrifolia*  (sunsunia),  *Salvia triloba* | Antioxidant, anti-inflammatory, anticholinesterase |
| 30 | Kaempferol | 5280863 | 286.23 | *Marsilea quadrifolia*  ( sunsunia) |  |
| 31 | Curcumin | 969516 | 368.38 | *Curcuma longa*  ( turmeric) | Antioxidant,  anti-inflammatory |
| 32 | Alpha-asarone | 636822 | 208.25 | *Acorus calamus* | Antioxidative, anticholinesterase |
| 33 | Beta-asarone | 5281758 |  |  |  |
| 34 | Eugenol | 3314 | 164.20 |  |  |
| 35 | Beta-cadinene | 10657 | 204.35 | *Ferula asafoetida* | Anti-COX-1 |
| 36 | Eremophilene | 12309744 |  |  |  |
| 37 | Elatin (flavonoid) | 44257938 | 594.52 | *Chamaecrista mimosoides*,  *Buddleja salviifolia*, *Salvia tiliifolia*, *Schotia brachypetala* (root, bark) | Antioxidative, anticholinesterase |
| 38 | Proanthocyanidins | 21881649, 108065 | 592.55 |  |  |
| 39 | Phenol | 110629 | 148.20 |  |  |
| 40 | Brassicasterol | 5281327 | 398.67 | *Brassica* species | Anti-inflamatory, neuroprotechtive, anticholinesterase |
| 41 | Sinapine | 5280385 | 310.37 |  |  |
| 42 | Sinapic acid | 637775 | 224.21 |  |  |
| 43 | Ginkgetin | 5271805 | 566.51 | *Ginkgo biloba* | Anticholinesterase |
| 44 | Ginkgolide A | 9909368 | 408.40 |  |  |
| 45 | Lupeol | 259846 | 426.72 | *Ptychopetalum olacoides* | Anticholinesterase |
| 46 | Beta-pinene | 14896 | 136.23 |  |  |
| 47 | Gingerol | 3473 | 294.39 | *Zingiber officinale* | Anti-COX-1 |
| 48 | 6-shogaol | 5281794 | 276.37 |  |  |
| 49 | Zingerone | 31211 | 194.23 |  |  |
| 50 | Piperitone | 6987 | 152.23 | *Cymbopogon schoenanthus* | Antioxidative, anticholinesterase |
| 51 | 2-carene | 79044 | 136.23 |  |  |
| 52 | Rosmarinic acid | 5281792 | 360.31 | *Salvia triloba* | Antioxidative, anticholinesterase |
| 53 | Ferulic acid | 445858 | 194.18 |  |  |
| 54 | Luteolin | 5280445 | 286.23 |  |  |
| 55 | Protopine | 4970 | 353.37 | *Corydalis ternate* | Anticholinesterase |
| 56 | Angelicin | 10658 | 186.16 | *Naradomansi jatamansi* | Antioxidative, anticholinesterase |
| 57 | Beta-eudesmol | 91457 | 222.37 |  |  |
| 58 | Calarene | 15560278 | 204.35 |  |  |
| 59 | Piperine | 638024 | 285.34 | *Piper nigrum* | Antioxidative, anticholinesterase |
| 60 | norharmane | 91737553 | 240.38 | *Peganum harmala* | Antioxidative |
| 61 | Harmine | 5280953 | 212.25 |  |  |
| 62 | Harmalol | 5353656 | 200.24 |  |  |
| 63 | Bacoside A | 92043183 | 768.98 | *Bacopa monnieri* | Anticholinesterase |
| 64 | Apigenin | 5280443 | 270.24 |  |  |
| 65 | Caravacrol | 10364 | 150.22 | *Origanum ehrenbergii*, *Origanum syriacum* | Anti-inflamatory, antioxidative, anticholinesterase |
| 66 | Thymol | 6989 | 150.22 |  |  |
| 67 | Diterpenoid EF-D | 198036 | 474.59 | *Salvia miltiorrhiza* | Anticholinesterase |
| 68 | Vasicine | 667496 | 188.23 | *Adhatoda vasica* | Anticholinesterase |
| 69 | Vasicol | 92470596 | 206.24 |  |  |
| 70 | Vasicinol | 442934 | 204.22 |  |  |
| 71 | Arachidic acid | 10467 | 312.53 |  |  |
| 72 | Cerotic acid | 44256495 | 399.71 |  |  |
| 73 | Oleic acid | 445639 | 282.46 |  |  |
| 74 | Beta-caryophyllene | 5281515 | 204.35 | *Syzygium aromaticum* | anti-COX-1 |
| 75 | Humulene | 5281520 | 204.35 |  |  |
| 76 | Alpha-amyrin | 73170 | 426.72 | *Tabernaemontana divaricata* | Anticholinesterase |
| 77 | Voafinidine | 101243262 | 328.45 |  |  |
| 78 | Beta-sitosterol | 222284 | 414.71 |  |  |
| 79 | Tanshinone IIA | 164676 | 294.35 | *Salvia miltiorrhiza* | Anticholinesterase |
| 80 | Cedrene | 521207 | 204.35 |  |  |

CID, compound ID; MW, molecular weight.
