# Supplementary material for: In silico discovery and evaluation of phytochemicals binding mechanism against human catechol-O-methyltransferase as a putative bioenhancer of L-DOPA therapy in Parkinson disease
Source: Genomics Inform. 2020 Dec 23;19(1):e7. doi: 10.5808/gi.20061 (PMC8042297; doi:10.5808/gi.20061)
Supplement: Supplementary Table 2. — Predicted drug likeness properties of total eighty phytochemicals [file gi-20061suppl2.docx]

**Supplementary Table 2.** Predicted drug likeness properties of total eighty phytochemicals

| No. | Ligand | Drug likeness properties | | | | | | | Remark |
| --- | --- | --- | --- | --- | --- | --- | --- | --- | --- |
|  |  | [miLogP](http://www.molinspiration.com/services/logp.html) | [TPSA](http://www.molinspiration.com/services/psa.html) | MW | nHBA | nHBD | nrotb | [Vol](http://www.molinspiration.com/services/volume.html) |  |
| 1 | 2-Carene | 3.45 | 0.00 | 136.24 | 0 | 0 | 0 | 151.81 | Suitable |
| 2 | 6-Shogaol | 4.35 | 46.53 | 276.38 | 3 | 1 | 9 | 281.38 | Suitable |
| 3 | Alpha-Asarone | 2.49 | 27.70 | 208.26 | 3 | 0 | 4 | 204.66 | Suitable |
| 4 | Anaferine | 1.38 | 41.12 | 224.35 | 3 | 2 | 4 | 236.41 | Suitable |
| 5 | Angelicin | 2.29 | 43.35 | 186.17 | 3 | 0 | 0 | 154.15 | Suitable |
| 6 | Apigenin | 2.46 | 90.89 | 270.24 | 5 | 3 | 1 | 224.05 | Suitable |
| 7 | Beta-Asarone | 2.49 | 27.70 | 208.26 | 3 | 0 | 4 | 204.66 | Suitable |
| 8 | Beta-Caryophyllene | 5.12 | 0.00 | 206.37 | 0 | 0 | 0 | 236.16 | Suitable |
| 9 | Beta-Eudesmol | 4.01 | 20.23 | 222.37 | 1 | 1 | 1 | 243.86 | Suitable |
| 10 | Beta-Pinene | 3.33 | 0.00 | 136.24 | 0 | 0 | 0 | 152.37 | Suitable |
| 11 | Calarene | 4.84 | 0.00 | 204.36 | 0 | 0 | 0 | 224.47 | Suitable |
| 12 | Calystegine B2 | ‒1.96 | 92.94 | 175.18 | 5 | 5 | 0 | 152.84 | Suitable |
| 13 | Caravacrol | 3.81 | 20.23 | 150.22 | 1 | 1 | 1 | 158.57 | Suitable |
| 14 | Cedrene | 4.76 | 0.00 | 204.36 | 0 | 0 | 0 | 224.47 | Suitable |
| 15 | Curcumin | 2.30 | 93.07 | 368.38 | 6 | 2 | 8 | 332.18 | Suitable |
| 16 | Cuscohygrine | 0.86 | 23.55 | 224.35 | 3 | 0 | 4 | 236.69 | Suitable |
| 17 | Diterpenoid EF-D | 4.26 | 110.14 | 474.59 | 7 | 2 | 7 | 448.34 | Suitable |
| 18 | Eugenol | 2.10 | 29.46 | 164.20 | 2 | 1 | 3 | 162.14 | Suitable |
| 19 | Furulic acid | 1.25 | 66.76 | 194.19 | 4 | 2 | 3 | 172.03 | Suitable |
| 20 | Genistein | 2.27 | 90.89 | 270.24 | 5 | 3 | 1 | 224.05 | Suitable |
| 21 | Gingerol | 3.22 | 66.76 | 294.39 | 4 | 2 | 10 | 295.61 | Suitable |
| 22 | Ginkgolide A | ‒1.46 | 128.60 | 408.40 | 9 | 2 | 1 | 339.84 | Suitable |
| 23 | Harmalol | 1.10 | 44.89 | 202.26 | 3 | 2 | 0 | 189.94 | Suitable |
| 24 | Harmine | 2.63 | 37.92 | 212.25 | 3 | 1 | 1 | 194.96 | Suitable |
| 25 | Kaempferol | 2.17 | 111.12 | 286.24 | 6 | 4 | 1 | 232.07 | Suitable |
| 26 | Luteolin | 1.97 | 111.12 | 286.24 | 6 | 4 | 1 | 232.07 | Suitable |
| 27 | Norharmane | 3.10 | 17.83 | 244.36 | 2 | 0 | 1 | 223.80 | Suitable |
| 28 | Pelletierine | 0.81 | 29.10 | 141.21 | 2 | 1 | 2 | 150.58 | Suitable |
| 29 | Phenol | 2.83 | 20.23 | 148.21 | 1 | 1 | 2 | 153.16 | Suitable |
| 30 | Piperine | 3.33 | 38.78 | 285.34 | 4 | 0 | 3 | 267.74 | Suitable |
| 31 | Piperitone | 3.20 | 17.07 | 152.24 | 1 | 0 | 1 | 165.13 | Suitable |
| 32 | Protopine | 2.75 | 57.24 | 353.37 | 6 | 0 | 0 | 308.00 | Suitable |
| 33 | Quercetin | 1.68 | 131.35 | 302.24 | 7 | 5 | 1 | 240.08 | Suitable |
| 34 | Sinapic acid | 1.26 | 76.00 | 224.21 | 5 | 2 | 4 | 197.57 | Suitable |
| 35 | Sinapine | ‒2.07 | 65.00 | 310.37 | 6 | 1 | 8 | 298.04 | Suitable |
| 36 | Tanshinone IIA | 4.16 | 47.28 | 294.35 | 3 | 0 | 0 | 269.89 | Suitable |
| 37 | Thymol | 3.34 | 20.23 | 150.22 | 1 | 1 | 1 | 158.57 | Suitable |
| 38 | Tropine | 0.48 | 23.47 | 141.21 | 2 | 1 | 0 | 146.00 | Suitable |
| 39 | Vasicine | 1.04 | 35.83 | 188.23 | 3 | 1 | 0 | 173.66 | Suitable |
| 40 | Vasicinol | 0.53 | 56.06 | 204.23 | 4 | 2 | 0 | 181.67 | Suitable |
| 41 | Vasicol | ‒0.02 | 66.56 | 206.25 | 4 | 3 | 2 | 191.51 | Suitable |
| 42 | Voafinidine | 2.44 | 48.63 | 328.46 | 4 | 2 | 1 | 321.43 | Suitable |
| 43 | Withafastuosin E | 2.65 | 124.29 | 488.62 | 7 | 4 | 4 | 460.49 | Suitable |
| 44 | WithaferinA | 3.86 | 96.36 | 470.61 | 6 | 2 | 3 | 442.38 | Suitable |
| 45 | Withanolide E | 3.18 | 116.59 | 486.61 | 7 | 3 | 2 | 449.16 | Suitable |
| 46 | WithanolideA | 4.15 | 96.36 | 470.61 | 6 | 2 | 2 | 441.81 | Suitable |
| 47 | WithanolideD | 4.15 | 96.36 | 470.61 | 6 | 2 | 2 | 441.81 | Suitable |
| 48 | Withanone | 4.15 | 96.36 | 470.61 | 6 | 2 | 2 | 441.81 | Suitable |
| 49 | Withaphysalin C | 3.92 | 102.30 | 484.59 | 7 | 2 | 1 | 444.80 | Suitable |
| 50 | Withaphysalin D | 2.82 | 89.91 | 466.57 | 6 | 1 | 1 | 429.95 | Suitable |
| 51 | Withaphysalin F | 3.43 | 105.59 | 484.59 | 7 | 2 | 1 | 440.22 | Suitable |
| 52 | Withaphysalin M | 2.29 | 102.44 | 482.57 | 7 | 1 | 1 | 434.36 | Suitable |
| 53 | Withaphysalin N | 2.31 | 102.44 | 484.59 | 7 | 1 | 1 | 440.55 | Suitable |
| 54 | Withasomnine | 2.66 | 17.83 | 184.24 | 2 | 0 | 1 | 176.23 | Suitable |
| 55 | Zingerone | 1.52 | 46.53 | 194.23 | 3 | 1 | 4 | 186.75 | Suitable |
| 56 | 28-Homocastasterone | 5.20 | 97.98 | 478.71 | 5 | 4 | 6 | 489.04 | Suitable |
| 57 | WithanolideB | 5.10 | 76.13 | 454.61 | 5 | 1 | 2 | 434.12 | Suitable |
| 58 | Withaphysalin O | 4.42 | 94.60 | 512.64 | 7 | 1 | 3 | 474.55 | Suitable |
| 59 | 24-Epibrassinolide | 5.12 | 107.22 | 480.69 | 6 | 4 | 5 | 481.23 | Suitable |
| 60 | Eremophilene | 5.01 | 0.00 | 204.36 | 0 | 0 | 1 | 229.95 | Suitable |
| 61 | Humulene | 5.30 | 0.00 | 204.36 | 0 | 0 | 0 | 234.00 | Suitable |
| 62 | Stigmasterol | ‒7.87 | 20.23 | 412.70 | 1 | 1 | 5 | 450.33 | Suitable |
| 63 | Withacnistin | 4.62 | 102.44 | 512.64 | 7 | 1 | 5 | 478.89 | Suitable |
| 64 | Daidzin | 0.77 | **149.82** | 416.38 | 9 | 5 | 4 | 348.15 | Not Suitable |
| 65 | Rosmarinic acid | 1.63 | **144.52** | 360.32 | 8 | 5 | 7 | 303.54 | Not Suitable |
| 66 | Alpha-Amyrin | **8.08** | 20.23 | 426.73 | 1 | 1 | 0 | 461.05 | Not Suitable |
| 67 | Arachidic acid | **8.73** | 37.30 | 312.54 | 2 | 1 | **18** | 358.63 | Not Suitable |
| 68 | Bacoside A | 3.11 | **215.83** | **771.00** | **13** | **8** | 13 | 740.86 | Not Suitable |
| 69 | Beta-cadinane | **5.97** | 0.00 | 204.36 | 0 | 0 | 1 | 229.75 | Not Suitable |
| 70 | Beta-Sitosterol | **8.62** | 20.23 | 414.72 | 1 | 1 | 6 | 456.52 | Not Suitable |
| 71 | Brassicasterol | **7.37** | 20.23 | 398.68 | 1 | 1 | 4 | 433.53 | Not Suitable |
| 72 | Cerotic acid | **9.63** | 37.30 | 396.70 | 2 | 1 | 24 | 459.44 | Not Suitable |
| 73 | Chlorogenic Acid | -0.45 | **164.74** | 354.31 | 9 | **6** | 5 | 296.27 | Not Suitable |
| 74 | Dulcitol | -3.10 | 121.37 | 182.17 | 6 | **6** | 5 | 161.66 | Not Suitable |
| 75 | Elatin (Flavonoid) | -1.58 | **271.19** | **594.52** | **15** | **11** | 4 | 486.12 | Not Suitable |
| 76 | Ginkgetin | **5.97** | **159.80** | **566.52** | 10 | 4 | 5 | 470.52 | Not Suitable |
| 77 | Lupeol | **8.29** | 20.23 | 426.73 | 1 | 1 | 1 | 461.60 | Not Suitable |
| 78 | Oleic acid | **7.58** | 37.30 | 282.47 | 2 | 1 | **15** | 318.84 | Not Suitable |
| 79 | Proanthocyanidins | 3.05 | **209.75** | **592.55** | **12** | **9** | 4 | 493.20 | Not Suitable |
| 80 | Scopoletin | 1.28 | 132.33 | 493.54 | **12** | 2 | 7 | 162.15 | Not Suitable |

Strongly deviation of any property was highlighted in bold font.

miLogP, Octanol/water partition coefficient; TPSA, topological polar surface area; MW, molecular weight; nHBA, number of hydrogen bond acceptors; nHBD, number of hydrogen bond donors; nrotb, number of rotatable bonds.
