# Supplementary material for: In silico discovery and evaluation of phytochemicals binding mechanism against human catechol-O-methyltransferase as a putative bioenhancer of L-DOPA therapy in Parkinson disease
Source: Genomics Inform. 2020 Dec 23;19(1):e7. doi: 10.5808/gi.20061 (PMC8042297; doi:10.5808/gi.20061)
Supplement: Supplementary Table 3. — Thirty-nine phytochemicals permeable through blood-brain barrier and their drug likeness properties [file gi-20061suppl3.docx]

**Supplementary Table 3**. Thirty-nine phytochemicals permeable through blood-brain barrier and their drug likeness properties

| No. | Ligand | Drug likeness properties | | | | | | | Remark |
| --- | --- | --- | --- | --- | --- | --- | --- | --- | --- |
|  |  | [miLogP](http://www.molinspiration.com/services/logp.html) | [TPSA](http://www.molinspiration.com/services/psa.html) | MW | nHBA | nHBD | nrotb | [Vol](http://www.molinspiration.com/services/volume.html) |  |
| 1 | 6-Shogaol | 4.35 | 46.53 | 276.38 | 3 | 1 | 9 | 281.38 | Suitable |
| 2 | Alpha-Asarone | 2.49 | 27.70 | 208.26 | 3 | 0 | 4 | 204.66 | Suitable |
| 3 | Anaferine | 1.38 | 41.12 | 224.35 | 3 | 2 | 4 | 236.41 | Suitable |
| 4 | Angelicin | 2.29 | 43.35 | 186.17 | 3 | 0 | 0 | 154.15 | Suitable |
| 5 | Beta-Asarone | 2.49 | 27.70 | 208.26 | 3 | 0 | 4 | 204.66 | Suitable |
| 6 | Beta-Caryophyllene | 5.12 | 0.00 | 206.37 | 0 | 0 | 0 | 236.16 | Suitable |
| 7 | Beta-Eudesmol | 4.01 | 20.23 | 222.37 | 1 | 1 | 1 | 243.86 | Suitable |
| 8 | Beta-Pinene | 3.33 | 0.00 | 136.24 | 0 | 0 | 0 | 152.37 | Suitable |
| 9 | Calarene | 4.84 | 0.00 | 204.36 | 0 | 0 | 0 | 224.47 | Suitable |
| 10 | Caravacrol | 3.81 | 20.23 | 150.22 | 1 | 1 | 1 | 158.57 | Suitable |
| 11 | Cedrene | 4.76 | 0.00 | 204.36 | 0 | 0 | 0 | 224.47 | Suitable |
| 12 | Cuscohygrine | 0.86 | 23.55 | 224.35 | 3 | 0 | 4 | 236.69 | Suitable |
| 13 | Eugenol | 2.10 | 29.46 | 164.20 | 2 | 1 | 3 | 162.14 | Suitable |
| 14 | Furulic acid | 1.25 | 66.76 | 194.19 | 4 | 2 | 3 | 172.03 | Suitable |
| 15 | Gingerol | 3.22 | 66.76 | 294.39 | 4 | 2 | 10 | 295.61 | Suitable |
| 16 | Harmalol | 1.10 | 44.89 | 202.26 | 3 | 2 | 0 | 189.94 | Suitable |
| 17 | Harmine | 2.63 | 37.92 | 212.25 | 3 | 1 | 1 | 194.96 | Suitable |
| 18 | Norharmane | 3.10 | 17.83 | 244.36 | 2 | 0 | 1 | 223.80 | Suitable |
| 19 | Pelletierine | 0.81 | 29.10 | 141.21 | 2 | 1 | 2 | 150.58 | Suitable |
| 20 | Phenol | 2.83 | 20.23 | 148.21 | 1 | 1 | 2 | 153.16 | Suitable |
| 21 | Piperine | 3.33 | 38.78 | 285.34 | 4 | 0 | 3 | 267.74 | Suitable |
| 22 | Piperitone | 3.20 | 17.07 | 152.24 | 1 | 0 | 1 | 165.13 | Suitable |
| 23 | Protopine | 2.75 | 57.24 | 353.37 | 6 | 0 | 0 | 308.00 | Suitable |
| 24 | Sinapic acid | 1.26 | 76.00 | 224.21 | 5 | 2 | 4 | 197.57 | Suitable |
| 25 | Sinapine | -2.07 | 65.00 | 310.37 | 6 | 1 | 8 | 298.04 | Suitable |
| 26 | Tanshinone IIA | 4.16 | 47.28 | 294.35 | 3 | 0 | 0 | 269.89 | Suitable |
| 27 | Thymol | 3.34 | 20.23 | 150.22 | 1 | 1 | 1 | 158.57 | Suitable |
| 28 | Tropine | 0.48 | 23.47 | 141.21 | 2 | 1 | 0 | 146.00 | Suitable |
| 29 | Vasicine | 1.04 | 35.83 | 188.23 | 3 | 1 | 0 | 173.66 | Suitable |
| 30 | Vasicinol | 0.53 | 56.06 | 204.23 | 4 | 2 | 0 | 181.67 | Suitable |
| 31 | Vasicol | -0.02 | 66.56 | 206.25 | 4 | 3 | 2 | 191.51 | Suitable |
| 32 | Voafinidine | 2.44 | 48.63 | 328.46 | 4 | 2 | 1 | 321.43 | Suitable |
| 33 | Withaphysalin D | 2.82 | 89.91 | 466.57 | 6 | 1 | 1 | 429.95 | Suitable |
| 34 | Withasomnine | 2.66 | 17.83 | 184.24 | 2 | 0 | 1 | 176.23 | Suitable |
| 35 | Zingerone | 1.52 | 46.53 | 194.23 | 3 | 1 | 4 | 186.75 | Suitable |
| 36 | WithanolideB | 5.10 | 76.13 | 454.61 | 5 | 1 | 2 | 434.12 | Suitable |
| 37 | Eremophilene | 5.01 | 0.00 | 204.36 | 0 | 0 | 1 | 229.95 | Suitable |
| 38 | Humulene | 5.30 | 0.00 | 204.36 | 0 | 0 | 0 | 234.00 | Suitable |
| 39 | Stigmasterol | ‒7.87 | 20.23 | 412.70 | 1 | 1 | 5 | 450.33 | Suitable |

miLogP, octanol/water partition coefficient; TPSA, topological polar surface area; MW, molecular weight; nHBA, number of hydrogen bond acceptors; nHBD, number of hydrogen bond donors; nrotb, number of routable bonds.
