# Supplementary material for: In silico discovery and evaluation of phytochemicals binding mechanism against human catechol-O-methyltransferase as a putative bioenhancer of L-DOPA therapy in Parkinson disease
Source: Genomics Inform. 2020 Dec 23;19(1):e7. doi: 10.5808/gi.20061 (PMC8042297; doi:10.5808/gi.20061)
Supplement: Supplementary Table 4. — Binding energy scores of 63 drug-like phytochemicals resulted from virtual screening against human COMT enzyme [file gi-20061suppl4.docx]

**Supplementary Table 4.** Binding energy scores of 63 drug-like phytochemicals resulted from virtual screening against human COMT enzyme

| No. | Phytochemical | Active site based virtual screening (kcal/mol) |
| --- | --- | --- |
| 1 | Withaphysalin M | ‒10.3 |
| 2 | Withaphysalin N | ‒10.3 |
| 3 | Withaphysalin F | ‒9.9 |
| 4 | Withaphysalin O | ‒9.5 |
| 5 | Protopine | ‒6.7 |
| 6 | Tanshinone IIA | ‒6.5 |
| 7 | Withafastuosin E | ‒6.3 |
| 8 | Luteolin | ‒6.3 |
| 9 | WithaferinA | ‒6.1 |
| 10 | Quercetin | ‒6.1 |
| 11 | Stigmasterol | ‒6.1 |
| 12 | WithanolideB | ‒6.0 |
| 13 | WithanolideD | ‒5.9 |
| 14 | Withacnistin | ‒5.9 |
| 15 | Withaphysalin C | ‒5.8 |
| 16 | Genistein | ‒5.8 |
| 17 | Kaempferol | ‒5.8 |
| 18 | Calarene | ‒5.8 |
| 19 | Curcumin | ‒5.7 |
| 20 | Beta-Eudesmol | ‒5.7 |
| 21 | WithanolideA | ‒5.5 |
| 22 | Withaphysalin D | ‒5.3 |
| 23 | Withasomnine | ‒5.3 |
| 24 | Eremophilene | ‒5.3 |
| 25 | Vasicinol | ‒5.3 |
| 26 | 6-Shogaol | ‒5.3 |
| 27 | Calystegine B2 | ‒5.2 |
| 28 | 24-Epibrassinolide | ‒5.2 |
| 29 | Piperine | ‒5.2 |
| 30 | Angelicin | ‒5.2 |
| 31 | Gingerol | ‒5.2 |
| 32 | Harmalol | ‒5.2 |
| 33 | Apigenin | ‒5.1 |
| 34 | Withanone | ‒5.1 |
| 35 | Withanolide E | ‒5.1 |
| 36 | Vasicine | ‒5.1 |
| 37 | Ferulic acid | ‒5.0 |
| 38 | Phenol | -5.0 |
| 39 | Harmine | ‒5.0 |
| 40 | Beta-Caryophyllene | ‒5.0 |
| 41 | Sinapine | ‒5.0 |
| 42 | Norharmane | ‒4.9 |
| 43 | Eugenol | ‒4.9 |
| 44 | Humulene | ‒4.9 |
| 45 | Zingerone | ‒4.9 |
| 46 | 2-Carene | ‒4.9 |
| 47 | Sinapic acid | ‒4.8 |
| 48 | 28-Homocastasterone | ‒4.7 |
| 49 | Voafinidine | ‒4.7 |
| 50 | Caravacrol | ‒4.7 |
| 51 | Thymol | -4.6 |
| 52 | Vasicol | ‒4.6 |
| 53 | Anaferine | ‒4.6 |
| 54 | Beta-Pinene | ‒4.6 |
| 55 | Alpha-Asarone | ‒4.5 |
| 56 | Piperitone | ‒4.5 |
| 57 | Cedrene | ‒4.5 |
| 58 | Diterpenoid EF-D | ‒4.4 |
| 59 | Ginkgolide A | ‒4.2 |
| 60 | Beta-Asarone | ‒4.2 |
| 61 | Cuscohygrine | ‒4.2 |
| 62 | Tropine | ‒4.0 |
| 63 | Pelletierine | ‒3.9 |
